# Supplementary material for: Adaptive chunking improves effective working memory capacity in a prefrontal cortex and basal ganglia circuit
Source: bioRxiv. 2024 Nov 21:2024.03.24.586455. Preprint. [Version 2] doi: 10.1101/2024.03.24.586455 (PMC11601399; doi:10.1101/2024.03.24.586455)
Supplement: Supplement 1 [file NIHPP2024.03.24.586455v2-supplement-1.pdf]

bioRxiv preprint doi: https://doi.org/10.1101/2024.03.24.586455; this version posted November 21, 2024. The copyright holder for this preprint (which was not certified by peer review) is the author/funder, who has granted bioRxiv a license to display the preprint in perpetuity. It is made available under aCC-BY 4.0 International license.

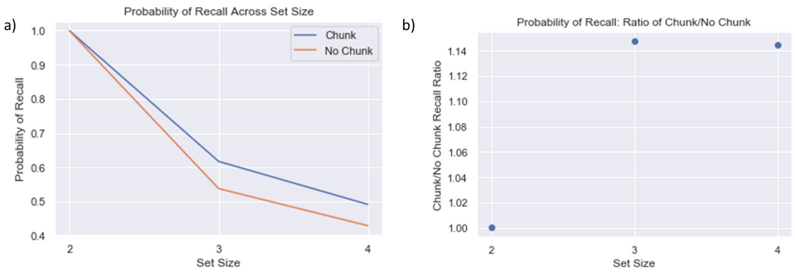

Figure 5—figure supplement 1. P(Recall) Across Set Size a) Average recall probability across set sizes decreases with set size, but less so for chunk models. Note that chance performance is approximately 19%. b) Chunk models have a higher ratio of recall probability relative to no chunk model when set size exceeds allocated capacity. This analysis includes trials where the variance across colors is low (standard deviation was < 35 degrees). The same chunk advantages would across all trials (including with high variance; not shown) but we focus on low variance trials wherein models can perform reasonably well even if accidentally mistaking one item for another (swap errors). Here we confirm that the chunk model improvement occurs over and above such effects.
